# Elemene sensitizes pancreatic cancer cells to bortezomib by enhancing proteasome inhibition via molecular patch mechanism

**DOI:** 10.1038/s41392-023-01373-z

**Published:** 2023-02-27

**Authors:** Shurong Hou, Zhenzhen Li, Xiaoling Chen, Wenxin Wang, Ting Duan, Louis Scampavia, Yaxia Yuan, Timothy P. Spicer, Xiabin Chen, Tian Xie

**Affiliations:** 1 School of Pharmacy, Key Laboratory of Elemene Class Anti-Cancer Chinese Medicines, Engineering Laboratory of Development and Application of Traditional Chinese Medicines, Collaborative Innovation Center of Traditional Chinese Medicines of Zhejiang Province, Hangzhou Normal University, Hangzhou, Zhejiang 311121 China; 2 Department of Pharmacology, School of Pharmacy, Nanjing University of Chinese Medicine, Nanjing, Jiangsu 210023 China; 3 UF Scripps Molecular Screening Center, Department of Molecular Medicine, UF Scripps Biomedical Research, Jupiter, FL 33458 USA; 4 Department of Biochemistry and Structural Biology, University of Texas Health Science Center at San Antonio, San Antonio, TX 78229 USA

**Keywords:** Drug screening, Drug development

**Dear Editor**,

Pancreatic cancer* is one of the deadliest cancers, with an overall 5-year survival rate of less than 10% unchanged over the last 40 years. Approximately 80–85% of patients are diagnosed as advanced stage, which are not eligible for curative surgery.^1^ Therapeutic options for advanced pancreatic cancer patients are still chemotherapy drugs with limited outcomes to improve patient survival and life quality.^2^ New insights into developing safe and effective drugs for this lethal disease are urgently needed. Elemene (Ele) is a sesquiterpene natural product extracted from the Chinese herb *Curcuma wenyujin*. Developed with the guidance of “molecular compatibility theory”,^3^ it is formulated as elemene liposome emulsion and approved by China’s Food and Drug Administration for treatment of some common cancers, including lung, liver, gastric, colorectal cancer and pancreatic cancer.^4,5^ More interestingly, studies reported that elemene produces combinative effects with some chemotherapies,^6,7^ such as oxaliplatin, gefitinib. Therefore, systematic exploration of the combined application of elemene with oncology drugs to achieve better therapeutic effects is of great clinical significance given the grim reality of pancreatic cancer treatment.

In this study, systematically searching for the potential combination agent with elemene was performed with high-throughput screening of approved oncology drugs in pancreatic cancer cells to identify the drugs that synergize with elemene. A class of proteasome inhibitors was identified, with the combination effect rank: bortezomib >ixazomib >carfilzomib (Supplementary Fig. S1). Bortezomib displayed the most potent combination effects with elemene, whereas carfilzomib did not demonstrate any combination effects with elemene. A low, non-toxic dose of elemene dramatically enhanced the cytotoxicity of bortezomib toward pancreatic cancer cells by 24 to 111-fold among 2D and 3D models (Fig. 1a–e). The coefficient of drug interaction (CDI) was calculated to further evaluate their combination effects according to the equation: CDI = AB/(A × B). Calculated CDI values for most concentrations that applied to all the four cell models in Fig. 1f demonstrate prominent synergistic effects between elemene and bortezomib (CDI < 0.75). These results indicate that elemene strongly synergizes with bortezomib in enhancing its cytotoxicity against pancreatic cancer cells.

**Fig. 1:**
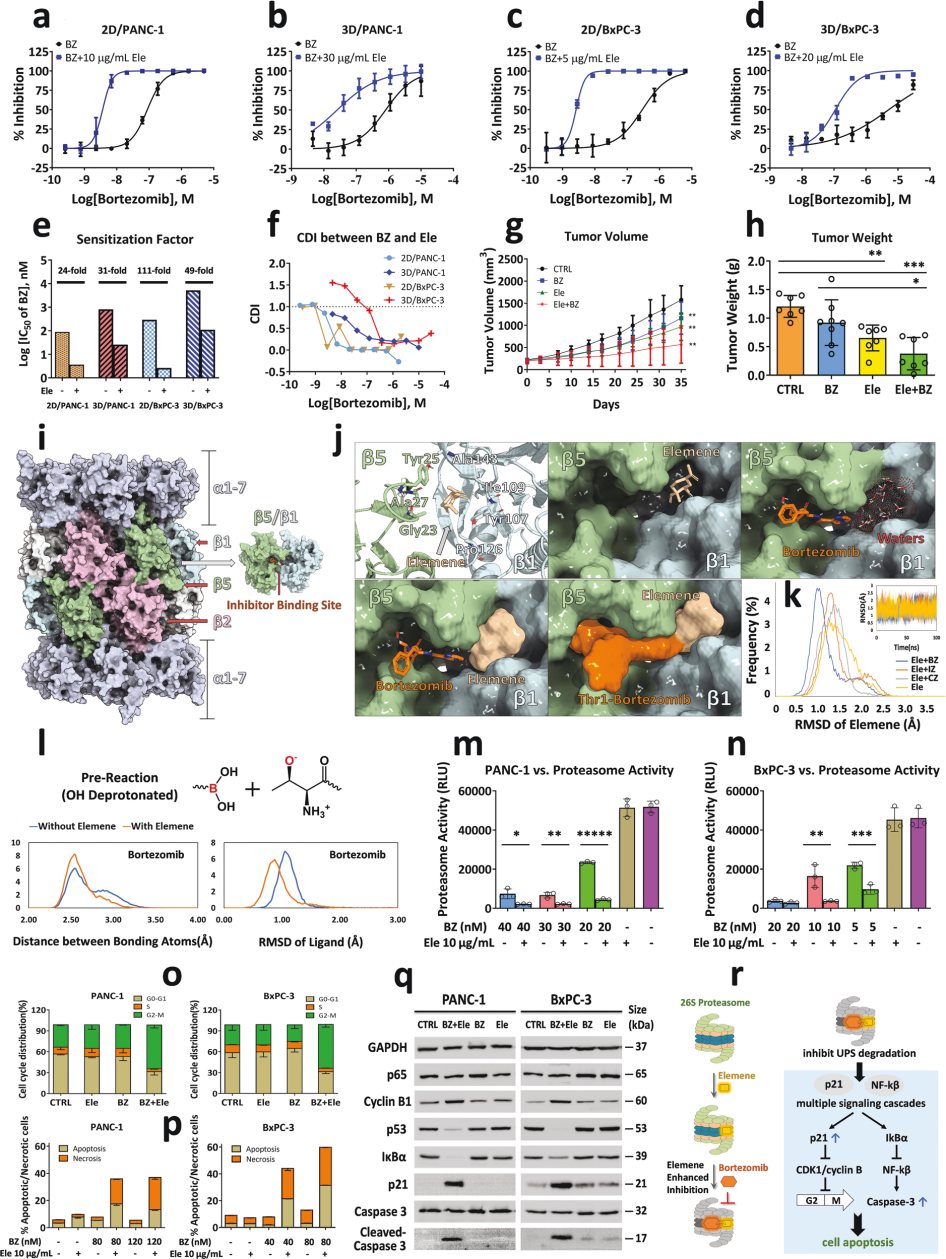
Elemene enhances bortezomib’s anti-pancreatic cancer efficacy by reinforcing proteasome inhibition via molecular patch mechanism. Elemene synergizes with bortezomib in enhancing the cytotoxicity of bortezomib against 2D and 3D pancreatic cancer cells (**a**–**f**). Concentration-response curves of bortezomib in the absence or presence of elemene in 2D/PANC-1 (**a**), 3D/PANC-1 (**b**), 2D/BxPC-3 (**c**), and 3D/BxPC-3 (**d**) cells (*n* = 3). Synergistic effects in terms of the sensitization factor of elemene to bortezomib (**e**) and calculated coefficient of drug interaction (CDI) between elemene and bortezomib (**f**) in tested four cell models. Antitumor efficacy of elemene and bortezomib in PANC-1 xenografts evaluated by tumor growth curves during treatment (**g**) and endpoint tumor weight (**h**) (*n* = 7). Computational modeling suggests elemene alters inhibitor binding profile by reshaping the ligand binding site of proteasome (**i**–**j**). **i** Structure of 20 S proteasome (PDB 5LF3). All α-subunits are colored in lightblue. β1, β2, and β5 subunits are colored in cyan, pink, and green respectively. Other β subunits are colored in white. **j** Modeled binding conformation of β1-β5-elmene, β1-β5-bortezomib, and β1-β5-bortezomib-elemene (the corresponding binding conformations for ixazomib and carfilzomib are depicted in Supplementary Fig. S4). Molecular dynamics simulations demonstrate the molecular patch mechanism of elemene in enhancing proteasome inhibition (**k**–**l**). **k** Histogram distribution of RMSD for elemene in presence of non-covalent binding bortezomib, ixazomib, carfilzomib, or in absence of inhibitor. The trajectories are aligned based on protein part. **l** Comparison of histogram distribution of RMSD and distance of bonding atoms with/without elemene binding in intermediate state (non-covalent binding state and covalent binding state are shown in Supplementary Fig. S5b). The trajectories are aligned based on protein part. The bonding atom distance for bortezomib is defined as the distance between boron atom and side chain oxygen atom of Thr1 in β5 subunit. Elemene enhances the inhibitory activity of bortezomib toward proteasome (**m**–**n**), and thus affects downstream regulators of cell cycle and apoptosis process (**o**–**q**). Elemene augments the inhibitory activity of bortezomib against cellular proteasome in PANC-1 cells (**m**) and BxPC-3 cells (**n**) (*n* = 3). **o** Cell cycle distribution of 2D/PANC-1 and 2D/BxPC-3 cells upon elemene and bortezomib treatment for 24 h (*n* = 3). **p** Apoptosis and necrosis after elemene and bortezomib treatment for 24 h determined by flow cytometry (*n* = 3). **q** Western blot analysis of cell cycle and apoptosis-associated key regulatory proteins in pancreatic cancer cells after drug treatment for 48 h. **r** A proposed schematic diagram of elemene’s role in enhancing bortezomib-induced cytotoxicity in pancreatic cancer cells via reinforcing proteasome inhibition and resultant cell cycle arrest and apoptosis induction

To further confirm above findings, the antitumor efficacy of bortezomib in combination with elemene was examined in a pancreatic cancer xenograft model. Compared with the control group, bortezomib or elemene alone inhibited the tumor growth to a certain extent, while the combination treatment significantly enhanced the antitumor activity relative to bortezomib alone assessed by tumor growth curve and endpoint tumor weight (Fig. 1g, h), suggesting that elemene boosts bortezomib’s anti-pancreatic cancer activity, and their combination displays significant antitumor efficacy with no obvious systematic toxicity (Supplementary Fig. S9a).

To explore the potential downstream mechanism for elemene’s synergism with bortezomib in inhibiting cell growth, a proteomic assay was first performed in PANC-1 cells treated with a single agent or their combination. Most of differentially regulated proteins are enriched in the common signaling pathways upon proteasome inhibition, including proteasome degradation, cell cycle, apoptosis and translation (Supplementary Fig. S3), indicating that proteasome inhibition-associated cell cycle and apoptosis pathway disturbance might be the mechanism by which elemene produces the synergistic antitumor effects with bortezomib. Computational modeling shows that elemene could stably bind to an unoccupied region nearby the bortezomib, ixazomib, and carfilzomib binding site in β5-β1 complex (Fig. 1i, j, Supplementary Fig. S4b). Further extensive MD simulation indicates that elemene could contact and stabilize nearby inhibitors, with facilitating the reaction of bond forming of bortezomib and ixazomib (Fig. 1k, l, Supplementary Fig. S5). So the small molecular elemene could enhance bortezomib and ixazomib binding via accelerating the forming of reversible bonds, and it could also stabilize the covalent binding state which may reduce the possibility of breaking of the reversible bonds. In contrast, elemene does not facilitate the bond forming of carfilzomib in binding state, which may explain the inefficacy of elemene in combination with carfilzomib (Supplementary Fig. S2b). Based on this observation, we propose a “molecular patch” mechanism (Supplementary Fig. S6) for explaining the mechanism of action of commonly believed non-specific low-molecular-weight compounds, which defines as a molecule binding, reshaping the original ligand binding region of target protein and consequently altering the binding profile of other molecules.

To validate whether elemene works synergistically with bortezomib in inhibiting cell growth by enhancing bortezomib’s action via proteasome inhibition, cellular proteasome activity was examined by Cell-based Proteasome-Glo Assays. Compared to the bortezomib group, addition of elemene significantly increased bortezomib-associated proteasome inhibition activity in both PANC-1 and BxPC-3 cells (Fig. 1m, n), whereas it did not present such effects with carfilzomib (Supplementary Fig. S2c). To confirm their binding of elemene and bortezomib with proteasome, cellular thermal shift assays (CETSA) were performed in PANC-1 and BxPC-3 cells. CETSA demonstrates elemene protects bortezomib-treated proteasome against thermal denaturation (Supplementary Fig. S7), which suggests that elemene stabilizes the binding of bortezomib with proteasome in pancreatic cancer cells. These experimental studies suggest that the computationally proposed mechanism is reasonable.

As a potent proteasome inhibitor, bortezomib’s antitumor mechanism of action is through proteasome inhibition to disturb cell cycle regulation and apoptosis process. The proteasome is a multi-subunit complex that is responsible for the degradation of the majority of intracellular proteins in eukaryotes, and plays a critical role in the regulation of proteins that control cell cycle and apoptosis process.^8^ In line with enhanced proteasome inhibition demonstrated above, elemene dramatically promotes bortezomib-induced cell cycle arrest and apoptosis-related cell death toward pancreatic cancer cells (Fig. 1o, p, Supplementary Fig. S8). Some proteasome-associated downstream regulators of G2/M arrest and apoptosis such as p53, p21, cyclin B1, IkBα, p65, and cleaved-caspase 3 were verified by WB (Fig. 1q) and IHC (Supplementary Fig. S9b). Differential expression of these key regulators correlates with observed G2/M-phase arrest and apoptotic effects, which are comparable with previous studies regarding the effects of proteasome inhibition.^9^ All the concatenated computational and experimental data suggests the role of elemene in producing the synergistic antitumor effects by enhancing proteasome inhibition probably through the molecular patch mechanism, and promoting resultant cell cycle arrest and apoptosis through downstream regulatory proteins (Fig. 1r).

To summarize, elemene is an anticancer drug known for its broad-spectrum antitumor effects and lower toxicity in patients, and has been widely used as adjuvant therapy for cancer treatment in clinic. Bortezomib is the first-generation proteasome inhibitor that was approved for multiple myeloma and mantle cell lymphoma. However, its long-term use may be limited by treatment-related toxicities, including neurological, hematological and gastrointestinal complications as the most common side effects.^10^ Herein, we have found this novel combination to be effective and practical, and propose the sensitization mechanism of action, by which elemene reinforces the antitumor efficacy of bortezomib against pancreatic cancer cells both in vitro and in vivo probably via molecular patch mechanism. As a proof of concept study, this provides the rationale of their combination in pancreatic cancer treatment. Extensive efficacy and toxicity of this combination deserves further evaluation in both preclinical and clinical setting. It is expected that this novel combination strategy could provide the benefit in enhancing antitumor efficacy and thus possibly minimizing the toxicity of bortezomib. The proposed “molecular patch” mechanism demonstrates the mechanism of action for such a tiny and simple compound as elemene, may provide clues for understanding, optimizing, and ultimately designing of molecular-patch-based low-molecular-weight drugs.

## Supplementary information

Clean Supplemental Material

## Data Availability

All data are available upon reasonable request to the corresponding author.
