# Supplementary material for: Elemene sensitizes pancreatic cancer cells to bortezomib by enhancing proteasome inhibition via molecular patch mechanism
Source: Signal Transduct Target Ther. 2023 Feb 27;8:87. doi: 10.1038/s41392-023-01373-z (PMC9971206; doi:10.1038/s41392-023-01373-z)
Supplement: Supplementary file 1 — Clean Supplemental Material [file 41392_2023_1373_MOESM1_ESM.docx]

**Supplemental Materials for**

**Elemene sensitizes pancreatic cancer cells to bortezomib by enhancing proteasome inhibition via molecular patch mechanism**

Shurong Hou^1,#^, Zhenzhen Li^1,#^, Xiaoling Chen^1,#^, Wenxin Wang^3,1^, Ting Duan^1^, Louis Scampavia^2^, Yaxia Yuan^4^, Timothy P. Spicer^2,*^, Xiabin Chen^1,*^, Tian Xie^1,*^

Correspondence to: Timothy P. Spicer (spicert@ufl.edu),

Xiabin Chen (xch226@hznu.edu.cn)

Tian Xie (tianxie@hznu.edu.cn)

**This PDF file includes:**

Materials and Methods

Supplementary Text

Supplementary Figures S1 to S9

# Materials and Methods

## **Chemicals and reagents**

Bortezomib (#S101315) and carfilzomib (#S285305) was purchased from Selleckchem (Houston, USA). Elemene emusion was from Dalian Jingang Medicine Company (Dalian, China). Antibodies: GAPDH (#2118S), p53 (#2527S), and IkBα (#4814T) were purchased from Cell Signaling Technology (Danvers, USA). APP-CTFα (#ab32136), NF-kB p65 (#ab32536), p21 (#ab109520) and cleaved-caspase 3 (#ab32042) were purchased from Abcam (Cambridge, UK). The 20S Proteasome β1 (#sc-374405), 20S Proteasome β5 (#sc-393931), caspase 3 (#sc-5603) and cyclin B1 (#sc-245) were obtained from Santa Cruz (Dallas, USA).

## **Cell lines and culture**

The human pancreatic cancer PANC-1 and BxPC-3 cell lines were purchased from ATCC. Both cell lines were authenticated using STR locus analysis by VivaCell Biosciences Ltd. (Shanghai, China). They were maintained in DMEM medium with 10% fetal bovine serum (FBS) at 37°C in a humidified atmosphere of 95% humidity and 5% CO_2_. Cells were cultured and passaged using a 1:3 or 1:6 subcultivation ratio 2 or 3 times per week in standard tissue culture flasks. They were harvested and utilized in this format for the purpose of the following testing, using the same media as described above.

## **High throughput screening**

A collection of NCI-approved oncology drug set of 114 compounds was obtained directly from the NCI and were screened for elemene’s combination agents at the UF Scripps Molecular Screening Center.

A 1536-well 2D cell viability assay using commercially available luminescence detection reagent CellTiter-Glo (# G7573, Promega, USA) was implemented. 200 cells in 4 μL of culture media were seeded in 1536-well plates (# 789173-F, Greiner Bio-One, Monroe, USA). After incubation of the assay plates overnight (~14 h), cells were treated with various concentrations of NCI drugs (10 nL, 0.02% DMSO) and fixed low concentration of elemene (10 μg/mL, 1 μL) for 72 h. Cell viability was assessed with 5 μL of CellTiter-Glo reagent. The ViewLux microplate reader (PerkinElmer, Waltham, USA) was used to quantitate luminescence signal. IC_50_ values of NCI drugs with or without elemene were determined by fitting the concentration–response curve data with a four-parameter variable-slope method in GraphPad Prism.

## **2D and 3D cell viability assay**

Cytotoxicity of elemene and bortezomib or carfilzomib was further evaluated in 384-well 2D cell viability, which is the same as 1536-well format with cell number and volume adjusted accordingly. The 3D viability assay use the CellTiter-Glo 3D (#G9683, Promega, USA) was implemented as previously described. 2500 cells in 20 μL of culture media were seeded in Corning 384-well spheroid culture plates (#3830, Corning, USA). After 48 h incubation of the assay plates to ensure spheroid formation, cells were treated with various concentrations elemene and bortezomib or carfilzomib (5 μL). Cell viability was assessed after 72 h of incubation with 25 μL of CellTiter-Glo 3D reagent. Following a 30-min incubation at room temperature, luminescence was quantified on a microplate reader (Spark, Tecan, Switzerland).

## **Cell cycle analysis**

Cell cycle distribution was assessed by flow cytometry. A density of 6×10^5^ cells were seeded in 6 cm dish and then treated with elemene, bortezomib and their combination for 24 h. The cells were collected, washed twice with PBS before they were fixed in 75% anhydrous ethanol at 4°C overnight. Then, cells were washed with PBS to remove residual ethanol followed by staining with cell cycle staining kit (#550825, BD, USA) After incubation of 30 min at room temperature in the dark, cell cycle distribution was analyzed by FACS Calibur Flow Cytometer (CytoFLEX S, Beckman Coulter, USA).

## **Cell apoptosis and necrosis by flow cytometer**

Cell apoptosis was detected using a Pharmingen Annexin V-FITC/PI Apoptosis Detection Kit (#AP101, Liankebio, China) following the manufacturer’s instructions. Briefly, cells were seeded at a density of 7 × 10^5^ cells per well in 6-well plate. After overnight incubation, cells were treated with different concentrations of bortezomib and elemene for 24 h or 48 h. All cells were collected and resuspended in 400 μL ice-cold binding buffer, followed by addition of 3 μL of Annexin V-FITC and 3 μL of PI. After incubation at room temperature for 15 min in the dark, samples were analyzed using a FACS Calibur flow cytometer (CytoFLEX S, Beckman Coulter, USA).

## **Real Time-Glo Cell apoptosis and necrosis assay**

2D and 3D cell apoptosis and necrosis was evaluated using the Real Time-Glo Annexin V Apoptosis and Necrosis kit (#JA1011, Promega, USA). 2500 PANC-1 cells in 20 μL media per well were seeded in white TC treated 384-well plates for 2D analysis or Corning 384-well spheroid plates for 3D analysis. After overnight incubation or spheroid formation, 5 μL of tested drugs were added following by immediate addition of 25 μL Real Time-Glo Annexin V Apoptosis and Necrosis reagents. The luminescence value (RLU) and fluorescence value (RFU, 485 nM Ex/520 nM Em) were monitored with a microplate reader at different time points from 0 h to 72 h. The final concentration of elemene and bortezomib was 10 μg/mL and 80 nM respectively for 2D analysis, and 30 μg/mL and 3 μM respectively for 3D analysis.

## **Proteasome activity assay**

Proteasome activity of elemene and bortezomib was evaluated using the Proteasome-Glo Cell-Based Assay (#G8660, Promega, USA) according to the manufacturer’s instructions. PANC-1 and BxPC-3 cells were seeded in 384-well TC-treated white plates at a density of 3000 cells in 20 μL media per well. Cells were treated with 5 μL of elemene (10 μg/mL), various concentrations of bortezomib and their combinations for 2 h in cell culture incubator. The plate was equilibrated at room temperature for 10 minutes, then add 25 μL of Proteasome-Glo Chymotrypsin-Like Cell-Based Reagent to each well and incubate at room temperature for 10 min. The luminescence value was measured with a Tecan microplate reader.

## **Molecular docking for elemene**

The structure of 20S proteasome complex with bortezomib (PDB 5LF3), ixazomib (PDB 5LF7), carfilzomib (PDB 4R67), and apo structure (PDB 5LE5) were modeled for their interactions with elemene. The binding site is defined as the catalytic site on the interface of proteasome β5-β1 subunits. Autodock Vina was employed to perform the molecular docking studies.[^1^](#_ENREF_1)^,^ [^2^](#_ENREF_2) Autodock Tools were used to process the complex structures by removing the crystal waters and other molecules.[^3^](#_ENREF_3) The exhaustiveness and model number were set to 256 to improve the sampling of elemene binding conformation. Other docking parameters were set to default. The docking box size was set to 24*20*18 (Å). Top 20 conformations of each docking (total 80 conformations for 4 system) were saved for further evaluation. Implicit solvent model molecular dynamic simulations were performed on each docking complex for 100ps after 10,000 steps of the energy minimization using Amber18 package.[^4^](#_ENREF_4) Molecular Mechanics/Generalized Born Surface Area (MM/GBSA) method[^5^](#_ENREF_5) were used to calculate the binding free energy affinity of elemene for the total 80 candidate conformation. The conformation of elemene with lowest binding energy in 4 systems are similar. To avoid potential influence of using different starting conformation for further molecular dynamic simulation studies, we used the elemene conformation with lowest binding energy from apo structure system as the starting conformation for following study.

## **Parameterization of compounds and modified residues**

The conformation and protonation status of bortezomib, threonine-bonded bortezomib, ixazomib, threonine-bonded ixazomib, carfilzomib, threonine-bonded carfilzomib, and elemene were calculated by OpenBabel.[^6^](#_ENREF_6) The partial charge of bortezomib, ixazomib, carfilzomib, elemene were calculated with RESP2 approach[^7^](#_ENREF_7) with Gaussian09[^8^](#_ENREF_8) and Multiwfn^[9](#_ENREF_9)^. Structure optimization and single point energy calculation were performed at B3LYP/def2SVP and B3LYP/def2TZVP level with GD3BJ correction, respectively. AmberTools were used to parameterize these compounds and modified residues. Because boron atom is not supported in GAFF2 forcefield, the bond, angle and dihedral parameter from previous study[^10^](#_ENREF_10) were used for of bortezomib, threonine-bonded bortezomib, ixazomib, and threonine-bonded ixazomib. The OH-group deprotonated form of N-terminal threonine is not stable in QM calculation because the protonated N-terminal amino tends to send a hydrogen atom to the deprotonated oxygen, so keyword “ModRedun” were added in Gaussian calculation to fix the bond between nitron in N-terminal amino and its neighboring hydrogens. The boron-oxygen bonds in threonine-bonded bortezomib and threonine-bonded ixazomib were also fixed by “ModRedun” keyword to avoid reversible boron-oxygen bond breaking in QM calculation.

## **Molecular dynamic simulations**

Total 19 complex systems were prepared and subjected to 100ns MD simulation, including: (1) Elemene binds with β1-β5 (β1-β5 subunits extract from apo structure of proteasome, PDB 5LE5); (2) Bortezomib binds with β1-β5 subunits, with or without elemene, in non-covalent bind, reaction intermediate, and covalent binding status (β1-β5 subunits extract from bortezomib bound structure of proteasome, PDB 5LF3). The initial conformation of protein and bortezomib in covalent binding status is extracted from X-ray structure. For the initial conformation of non-covalent status, bond between sidechain oxygen of Thr-1 from β5 subunit and boron of bortezomib was manually removed, and an addition hydrogen were added to the sidechain oxygen of Thr-1. The parameter of deprotonated Thr-1 was generated by previous parametrization step. For the initial conformation of reaction intermediate status, bond between sidechain oxygen of Thr-1 from β5 subunit and boron of bortezomib was manually removed, without adding hydrogen to sidechain oxygen of Thr-1 to present the OH-group deprotected threonine. The parameter of bortezomib-modified Thr-1 were generated by previous parametrization step; (3) Ixazomib binds with β1-β5 subunits, with or without elemene, in non-covalent bind, reaction intermediate, and covalent binding status (β1-β5 subunits extract from ixazomib bound structure of proteasome, PDB 5LF7). The preparation of initial conformation is similar to bortezomib system. (4) Carfilzomib binds with β1-β5 subunits, with or without elemene, in non-covalent bind, reaction intermediate, and covalent binding status (β1-β5 subunits extract from carfilzomib bound structure of proteasome, PDB 4R67). The preparation of initial conformation is similar to bortezomib system. For the non-covalent and reaction intermediate status, the structure of reaction involved region of carfilzomib in the initial conformation were manually rebuilt according to energy minimized structure of carfilzomib. tLeap module of AmberTools were used to adding missing atoms. The protonation status of residues in protein were calculated with PDB2PQR.[^11^](#_ENREF_11) All molecular dynamics (MD) simulation were performed with Amber18 package.[^4^](#_ENREF_4) Threshold of non-boned interaction cutoff was set to 10Å, and the particle mesh Ewald (PME) was used to handle long-range electrostatic interactions. FF12SB and GAFF2 forcefield were used for protein and ligand part, respectively. OPC waters were used to build solvent box with adding 0.15 M KCl to mimic the physiological salt concentration. Total 100ns production simulation in NPT ensemble were performed after 10,000 steps of energy minimization, and 1ns equilibration simulation. The full 20S proteasome complex is too large for long time MD simulation, so only the β1-β5 subunits complex were extract from the original structure of proteasome complex. To avoid the significant conformational change of β1-β5 subunits complex without contacting with other subunits, all protein backbone atoms were fixed with a force constant of 100 kcal/mol/Å^2^ in all stages of simulation, while all the side chain atoms and ligand part were not constrained.

## **Cellular thermal shift assay**

The interaction of elemene and bortezomib with proteasome was evaluated in PANC-1 and BxPC-3 cells by Cellular thermal shift assay (CETSA). The thermostability of proteasome β5 subunit and β1 subunit which contains the binding site of bortezomib and elemene respectively, were assessed by western blot. Briefly, PANC-1 and BxPC-3 cells were cultured and grown until 70-80% confluency. Cells were detached with trypsin, collected by centrifugation, washed with PBS and subsequently resuspended in DMEM media for counting. Then exactly the same number of cells were seeded in 6 cm dishes and exposed to elemene (10 μg/mL), bortezomib (10 nM for PANC-1, 5 nM for BxPC-3) and their combination for 4 h at 37°C. After treatment, the whole cell pellet was aliquoted into PCR tubes and heated for 3 min at a temperature range (65-85°C). Subsequently, the cells were lysed by three repeated freeze-thawing cycles using liquid nitrogen followed by centrifugation at 14,000 rpm at 4°C to collect supernatant. Protein samples were mixed with loading buffer, boiled at 70 °C for 10 min, separated on 10% or 14% SDS-polyacrylamide gels, and transferred to polyvinylidene fluoride (PVDF) membranes. After blocking, the membranes were incubated with a primary antibody (20S proteasome β5 and β1, APP-CTFα as the loading control) overnight at 4°C, followed by washing with TBST before the incubation of HRP-conjugated secondary antibody for 1 h at room temperature. After through wash, the antibody associated protein bands were revealed using the Ncm ECL Ultra Western blotting kit (#P10100, Ncm Biotech, China), and visualized using the Touch Imager XLi (e-BLOT, China).

## **Proteomics analysis**

About 2×10^6^ cells were seeded in 10 cm dishes and treated with 10 μg/mL elemene, 40 nM bortezomib and their combination for 48 h. Total cells were collected and washed three times by cold PBS, and stored at -80℃ for LC-MS analysis. After trypsin digestion, label-free peptides were analyzed using an EASY-nLC 1000 liquid chromatography system (Thermo Fisher Scientific, USA) coupled to a LTQ Obitrap ETD mass spectrometer (Thermo Fisher Scientific, USA). Peptides were separated on a C18 column (1.8 mm, 0.15×100 mm) using a gradient elution. Raw MS files were analyzed by MaxQuant software (Max Planck Institute of Biochemistry, Germany).

The heatmap showing the relative abundance change of the indicated protein groups between different treatment groups in total PANC-1 cell lysate was based on normalized Log_2_ spectral abundance signal. The signal was averaged from the highly correlated datasets obtained in three independent experiments. For each given gene list, pathway and process enrichment analysis was conducted by Metascape^[12](#_ENREF_12)^ with the following ontology sources: GO Biological Processes, GO Cellular Components, Canonical Pathways and CORUM. Enriched terms with the top p values from up-regulation and down-regulation protein groups were selected.

## **Western blotting**

Pancreatic cancer cells were treated with elemene, bortezomib and their combination for 48 h. Total cell lysate was prepared with RIPA buffer (#P0013B, Beyotime, China). After centrifugation at 14,000 rpm at 4°C, the supernatant protein concentration was quantified by BCA protein assay (#P0010, Beyotime, China). Protein extraction kit (#P0028, Beyotime, China) was used to extract nuclear protein for p65 blotting. Protein samples were separated on SDS-polyacrylamide gels and transferred to polyvinylidene fluoride (PVDF) membranes. After blocking in 5% skimmed milk for 1.5 h, the membranes were incubated with a primary antibody overnight at 4°C, followed by washing with TBST before the incubation of HRP-conjugated secondary antibody for 1 h at room temperature. After through wash, the antibody associated protein bands were revealed using the Ncm ECL Ultra Western blotting kit (#P10100, Ncm Biotech, China), and visualized using the Touch Imager XLi (e-BLOT, China).

## ***In vivo* antitumor efficacy**

BALB/c nude mice (female, 6-8 weeks old) were purchased from GemPharmatech (Jiangsu, China) and maintained in a controlled environment under standard conditions in experimental animal facility at Hangzhou Normal University. The animal care and experimental protocols were approved by the animal care committee of Hangzhou Normal University. 3 × 10^6^ PANC-1 cells were subcutaneously injected into the right dorsal flank of BALB/c nude mice. Tumor volume was assessed twice a week and was calculated by the following formula: V = 0.52 × (L × W^2^), where L and W stands for two perpendicular diameters, *i.e.* the length and width of the tumors. The mice bearing the tumor range of 100-200 mm^3^ were randomly divided into four groups (7 mice per roup): (1) control group, treated with intraperitoneal injection of saline; (2) elemene group, treated with intraperitoneal injection of 40 mg/kg elemene emulsion, 6 times a week; (3) bortezomib group, treated with intraperitoneal injection of 0.6 mg/kg bortezomib, twice a week; (4) the combination group, treated with elemene and bortezomib, each drug schedule the same as single treatment group. Tumor growth and body weight of mice were evaluated twice a week. After 5 weeks of drug administration, the mice were sacrificed. The tumor tissues were resected and weighted followed by fixation in 10% neutral-buffered formalin for 24 h and embedded in paraffin.

## **Immunohistochemistry (IHC) staining**

Consecutive 5-μm thick slides of tumor tissues were sectioned for IHC staining. The slides were deparaffinized in xylene and rehydrated with graded ethanol to distilled water. Antigen retrieval was performed by boiling the slides in sodium citrate buffer for 2-3 min in the pressure boiler and wait until it cool down. The slides were incubated in 3% hydrogen peroxide for 20 min at room temperature to block the endogenous peroxidase activity. After washing with PBS, the slides were incubated with primary antibody overnight at 4°C in a moist chamber. Slides were then washed with PBS and subsequently incubated with Max Vision HRP Kits (#KIT-5004, Maixin, China) at room temperature for 30 min. Color was developed using 3,3′-diaminobenzidine (DAB) substrate kit (#DA1010, Solarbio, China). Hematoxylin (#G1120, Solarbio, China) was counterstained for nucleic acids. The slides were imaged using optical microscope (LX73, Olympus, Japan). ImageJ with IHC Toolbox was employed to calculate the %positive area of p21 and cleaved-caspase 3, using 4 field images per mouse and 4 mice per treatment group.

## **Statistical analysis**

GraphPad Prism 8.0 and SPSS 17.0 were used for statistical analysis. The comparison between groups were analyzed by student’s t-test or one-way ANOVA. Data were expressed as mean ± SD of three replicates, and statistically different values were considered for p-value < 0.05 (*p < 0.05, **p < 0.01, and ***p < 0.001).

## **Molecular visualization and operation**

Pymol 2.5.0 were used to operate and visualize structures.

# Supplementary Text

## **High throughput screen identifies bortezomib as elemene’s combination agent toward pancreatic cancer cells**

To systematically explore the combination agents of elemene, 114 NCI oncology drugs were tested in 1536-well plates against PANC-1 cells, as 10-points, threefold serial dilutions starting from a 5 μM nominal concentration, in the presence or absence of elemene. Based on the concentration-response curve (CRC) of elemene against PANC-1 cells in **Figure S1a**, the IC_50_ of elemene was determined to be 23 μg/mL. Elemene at 10 μg/mL produced ~5% inhibition, thus 10 μg/mL was chosen as the fixed concentration of elemene in the screen. IC_50_ values of each oncology drug in the presence or absence of elemene were compared to identify drugs that show the combination effects with elemene. The drug that displays a fold difference in IC_50_ value (*i.e.* IC_50 without ele_/IC_50 with ele_ defined as sensitization factor) larger than 2, was identified as the hit.

The screen statistics for 9 plates of 1536-well plates against PANC-1 cells: Z’ = 0.56±0.05; S/B = 120.5±8.0, respectively. One class of drugs emerged as the most interesting hits from this screening effort, *i.e.* bortezomib, ixazomib and carfilzomib known as proteasome inhibitors. As shown in **Figure S1b**, differential responses of bortezomib and ixazomib in the presence or absence of elemene were observed, and both of them become more cytotoxic to PANC-1 cells when combined with as low as 10 µg/mL elemene. However, no significantly different response of carfilzomib was observed with or without elemene. The IC_50_ values of proteasome inhibitors in the absence and presence of elemene were 1.64 nM and 0.55 nM for bortezomib, 2.48 μM and 1.21 μM for ixazomib, and 1.13 nM and 1.0 nM for carfilzomib, respectively. Screen results showed that elemene significantly enhanced the cytotoxicity of bortezomib against PANC-1 cells, while it did not present such an effect with carfilzomib. A slightly enhanced cytotoxicity of ixazomib was also achieved in the presence of elemene, but its IC_50_ value is 2200-fold higher than bortezomib. Therefore, ixazomib was not pursued in following experimental study.

Moreover, since bortezomib, ixazomib, and carfilzomib are all selective inhibitors that target proteasome and regulate the exact same biology pathway,[^13-15^](#_ENREF_13) their significantly different response in combination with elemene suggests that effect of elemene is dependent on the structure or inhibitory mechanism of these inhibitors. Especially, the complete inefficacy of elemene in combination with carfilzomib indicates that potential impact of elemene binding to any other proteins may be disregarded in this study.

## **Elemene synergizes with bortezomib in enhancing the cytotoxicity of bortezomib in both 2D and 3D pancreatic cancer cells**

To validate the results obtained from screening, 2D and 3D models of PANC-1 and BxPC-3 cells were employed to test their combination effects on cell growth inhibition by CellTiter-Glo assay. A spheroid-based 3D culture format that is better recapitulating the features of *in vivo* tumor,[^16^](#_ENREF_16) was used to verify the effects. Firstly, the concentration response of elemene against each model was determined to find an appropriate concentration without generating apparent cytotoxicity. As shown in **Figure S2a**, elemene at 5 or 10 µg/mL is appropriate for 2D assays, and 20 or 30 µg/mL for 3D assays. Comparing the concentration-response curves of bortezomib with and without elemene (**Figure 1a-d**), the pancreatic cancer cells became more sensitive to bortezomib with elemene addition. The sensitization factor of elemene to bortezomib based on their IC_50_ values toward 2D/PANC-1 and 2D/BxPC-3 was 24.2 and 111.4, respectively. A similar result was also observed in 3D/PANC-1 and 3D/BxPC-3, with the sensitization factor to be 31.4 and 49.4, respectively (**Figure 1e**). The dose response curves of carfilzomib, with and without elemene, overlapped with each other (**Figure S2b)**, and the corresponding sensitization factors of elemene to carfilzimib were all less than 2.

To evaluate the combination effects between elemene and bortezomib toward above tested pancreatic cancer cell models, the coefficient of drug interaction (CDI) was calculated according to the equation: CDI=AB/(A×B).[^17^](#_ENREF_17) As expected from the results above, calculated CDI values in **Figure 1f** demonstrated synergistic effects between elemene and bortezomib across a wide concentration range (CDI < 1). In addition, the CDI values for most concentrations that applied to all the four cell models were consistently < 0.75, which suggests a prominent synergistic effect. These results indicate that elemene strongly synergizes with bortezomib in enhancing its cytotoxicity against pancreatic cancer cells.

## **Elemene enhances the antitumor efficacy of bortezomib in a xenograft model**

To further confirm above findings, the antitumor efficacy of bortezomib in combination with elemene was examined in a pancreatic cancer xenograft model. PANC-1 cells were implanted into BALB/c nude mice subcutaneously and the tumor-bearing mice were further subjected to four different treatments (control, elemene, bortezomib, elemene plus bortezomib) when tumors reached 100 mm^3^ in size. The given schedule and dose for elemene and bortezomib treatment was designed according to their clinical use. Tumor growth and body weight of mice were evaluated twice a week. Bortezomib or elemene alone inhibited the tumor growth to a certain extent, while the combination treatment significantly enhanced the anti-tumor activity relative to bortezomib alone assessed by tumor growth curve and endpoint tumor weight. Compared with the control group, the endpoint tumor volume and weight was suppressed by 26.31% and 22.21% by bortezomib treatment, 38.56% and 45.83% by elemene treatment, and 68.04% and 68.64% by combined treatment of elemene and bortezomib, respectively (**Figure 1g & h**). Concurrently, mice body weight during treatment period was similar among all treatment groups (**Figure S9a**). These results suggest that elemene boosts bortezomib’s anti-pancreatic cancer activity, and their combination displays significant antitumor efficacy with no obvious systematic toxicity.

## **Proteomic analysis suggests that elemene synergizes with bortezomib through regulation of proteasome inhibition-associated cell cycle and apoptosis pathway**

To explore the potential downstream mechanism for elemene’s synergism with bortezomib in inhibiting cell growth, a proteomic assay was performed in PANC-1 cells treated with a single agent or their combination, and pathway enrichment analysis was applied to proteins with differential expression between the bortezomib group and bortezomib plus elemene group to define elemene’s effect. Addition of elemene to bortezomib treatment resulted in 631 up-regulated proteins and 279 down-regulated proteins, respectively (**Figure S3a**). Up-regulated proteins were enriched in the cell cycle, G2/M transition, programmed cell death, apoptosis, NF-kB signaling, and MAPK6/MAPK4 signaling pathway. Down-regulated proteins were enriched in the protein translation, cell cycle, G2/M transition, and transcriptional regulation by TP53 pathway. Most of differentially regulated proteins were enriched in the common signaling pathways upon proteasome inhibition, including proteasome degradation, cell cycle, apoptosis and translation as shown in the heatmap in **Figure S3b**. Moreover, these differentially expressed regulatory proteins could cause cell-cycle arrest and induce apoptotic-related cell death. These analysis suggests that proteasome inhibition-associated cell cycle and apoptosis pathway disturbance might be the mechanism by which elemene produces the synergistic antitumor effects with bortezomib.

## **Computational modeling suggests elemene alters inhibitor binding profile by reshaping the ligand binding site of proteasome**

Human 20S proteasome is composed of 14 α-subunits and 14 β-subunits arranged into four packed seven-subunit-rings,[^18^](#_ENREF_18) among which only the β1, β2, β5 are catalytic active subunits with caspase-like, trypsin-like, and chymotrypsin-like activity, respectively. It is reported that bortezomib, ixazomib, carfilzomib could inhibit β1, β2, and β5 subunits of proteasome, but all the three compounds preferentially inhibit β5 subunits.[^19^](#_ENREF_19) Considering the β5 is the dominate binding site of the three inhibitors, only the β5 subunit and its neighboring β1 subunits (**Figure 1i**) were considered in following computational study. As depicted in **Figure 1j** and **Figure S4b-c**, all the three inhibitors could form a covalent bond with the N-terminal threonine of β5, and occupy similar binding region between the interface of β5 and β1 subunits. To investigate the potential mechanism of elemene in enhanced proteasome inhibition, molecular docking was performed to predict the binding modes of elemene in β5-β1 complex, with or without the presence of inhibitor. The docked conformation with lowest binding free energy indicates that elemene is favorable to bind with a hydrophobic pocket that is not occupied by all the three inhibitors. Take the bortezomib binding mode as an example (**Figure 1j**), the assumed elemene binding region would be fulfilled with solvent molecules if elemene is not present. Elemene binding would repeal the solvent and decrease the solvent exposure of hydrophobic ring moiety of bortezomib and increase the hydrophobic contacts, which is expected to be in favor of bortezomib binding. From the perspective of binding site, elemene could be considered as a non-native cofactor of the β5-β1 complex which is involved in the forming of ligand binding site. As depicted in **Figure 1j** and **Figure S4b-c**, elemene binding could reshape the ligand binding site by fulfilling part of the unoccupied region and resulting in changed biophysical properties and interactions, which may alter the inhibitor binding profile. Furthermore, the computational model suggests that elemene is not competitive with three investigated inhibitors, which is consistent with the experimental results in **Figure 1m-n** that elemene does not affect the proteasome activity to chymotrypsin-like substrate. This model also supports the experimental observation that elemene enhanced bortezomib and ixazomib-induced cytotoxicity in **Figure S1b**. However, the inefficacy of elemene in combination with carfilzomib should be further analyzed.

## **Molecular dynamics simulations demonstrate the molecular patch mechanism of elemene in enhancing proteasome inhibition**

As depicted in **Figure S5a**, both bortezomib and ixazomib are reversible, boronic acid-based proteasome inhibitors, whereas carfilzomib is an irreversible, epoxyketone proteasome inhibitor.[^20^](#_ENREF_20) So, it would be straightforward to speculate the different response of bortezomib/ixazomib and carfilzomib are attributed to the different reaction mechanism. To investigate the influence of elemene on reaction, MD simulation was performed on three typical reaction states including non-covalent binding state (before reaction), pre-reaction state (ready for reaction), and covalent binding state (after reaction). The effect of elemene in combination with inhibitors in different states were analyzed by comparing the binding stability of inhibitors and distance of bond forming atoms in inhibitors and protein during MD simulation. It should be noted the term “pre-reaction state” in this study is not same with the intermediate state or transition state in chemical reaction that must be calculated at quantum chemistry level. The “pre-reaction state” is constructed by introducing N-terminal threonine OH-group deprotonation, which is a slow but essential step for inhibitors forming covalent bonds. So, the MD simulation for “pre-reaction state” that sampling the distance between bond forming atoms in inhibitor and the deprotonated threonine could be used to roughly estimate the possibility for starting the reaction. The stability of elemene during MD simulation was firstly investigated and demonstrated in **Figure 1k**. Although elemene is a low-molecular-weight compound, the binding conformation is very stable in 100 ns simulation, for apo β5-β1 complex and all β5-β1-inhibitor complexes. The peak of RMSD histogram map for all trajectories are below 1.5Å, which suggests high stability of elemene in the predicted binding site, especially for such a small molecule. The analysis of MD simulation in 100 ns trajectories all three states with or without the presence of elemene is shown in **Figure S5b**. For non-covalent binding state, bortezomib and ixazomib shows significant smaller distance for bond forming atoms in presence of elemene, which suggests that elemene could help the boron atom approaching the N-terminal threonine. In contrast, elemene does not facilitate carfilzomib forming the covalent bond. However, the left-shifted distribution of RMSD for three inhibitors with elemene binding indicates that elemene could stabilize the binding of inhibitors. For pre-reaction state, the elemene binding shows influence that is similar to the non-covalent binding state. For the covalent binding state, the bond is already forming so only the stability is evaluated. Similarly, elemene binding enhances the stability of all inhibitors. In summary, the binding of elemene could help to stabilize the inhibitor binding, but only facilitate the reaction for bortezomib and ixazomib. The structural influence of elemene binding on bortezomib, ixazomib, and carfilzomib complex were further demonstrated in **Figure S5c**. Bortezomib and ixazomib contains large hydrophobic moiety near the elemene binding region, which results in more contact-induced conformation change. Particularly, elemene could push the boron atoms in bortezomib and ixazomib approaching the hydroxyl group in Thr1 as well as forming hydrogen bond between boron neighboring oxygen and protonated amino group of Thr1, which is close to the transition state of the reaction. Bortezomib and ixazomib shows significantly different hydrogen bond forming in absence of elemene. The hydrogen bond between boron neighboring oxygen and hydroxyl group in Thr1 is expected to result in higher energy barrier for further boron-Thr1 reaction. In contrast, carfilzomib does not show different hydrogen bond forming with or without elemene binding. This could be explained by smaller hydrophobic moiety near elemene binding region for carfilzomib, comparing with bortezomib and ixazomib. The relatively less contacts between carfilzomib and elemene only slightly increase the stability of carfilzomib binding, but it is not sufficient to induce significant conformation change to facilitate the reaction. It should be noted that all the analysis is based on MD simulation for “inhibitor binding” status. The increased stability of carfilzomib is also expected to slightly facilitate the overall reaction possibility of carfilzomib to some content, although it is not expected to significantly facilitate the reaction in binding state.

In summary, bortezomib and ixazomib are expected to show better inhibition effect in presence of elemene based on the modelling, which is consistent with the following experimental observations. In contrast, elemene binding may have a weak effect for carfilzomib due to lack of reaction facilitation, even if elemene could stabilize carfilzomib to some content. Besides, carfilzomib is an irreversible inhibitor which will reach the maximal inhibition along with time, whose activity is less relevant to the binding affinity comparing with reversible inhibitors. So the relatively weak effect of elemene to carfilzomib may not be observed in 72 h long-incubation assay. This may explain the inefficacy of elemene in combination of carfilzomib. All the MD simulations show that elemene binding could influence the status of other ligands in the same binding region. Here we term this mechanism as “molecular patch” (**Figure S6**), which defines as a molecule binding, reshaping the original ligand binding region of target protein and consequently altering the binding profile of other molecules.

## **Elemene synergizes with bortezomib by stabilizing the binding of bortezomib with proteasome and thus enhancing proteasome inhibition**

To validate whether elemene works synergistically with bortezomib in inhibiting cell growth by enhancing bortezomib’s action via proteasome inhibition, cellular proteasome activity was examined by Cell-based Proteasome-Glo Assays. PANC-1 and BxPC-3 cells were treated with varying concentrations of bortezomib with or without 10 or 5 μg/mL elemene for 2 h. As expected, elemene alone did not display any inhibition, and bortezomib alone showed dose-response inhibition toward CT-like proteasome activity (**Figure 1m & n**). Compared to the bortezomib group, addition of elemene significantly increased bortezomib-associated proteasome inhibition activity by 36.9% in PANC-1 cells (from 54.9% to 91.8% in 20 nM group), and by an average of 27.7% in BxPC-3 cells (from 51.7% to 78.8%, 63.9% to 92.1% in 5 & 10 nM group). In contrast, no significant difference in proteasome inhibition was observed between carfilzomib group and carfilzomib plus elemene group in both PANC-1 and BxPC-3 cells (**Figure S2c**).

To further confirm their binding of elemene and bortezomib with proteasome in pancreatic cancer cells, cellular thermal shift assays (CETSA) were performed. Both PANC-1 and BxPC-3 cells were incubated with elemene and/or bortezomib for 4 h at 37°C, and subsequently subjected to increasing temperatures from 65°C to 85°C. The stability of both proteasome β5 subunit and β1 subunit which contains the binding site of bortezomib and the predicted binding site of elemene respectively, were assessed by western blot. GAPDH was found to degrade after 65°C, so thermostable APP-CTFα was used as the loading control.[^21^](#_ENREF_21) As suggested by Western blot analysis (**Figure S7**), although the protein level of APP-CTFα is low in PANC-1 cells, it was well detected in BxPC-3 cells. Thermal denaturation of proteasome β5 and β1 subunit in CTRL cells occurred at 70°C. The order of β5 and β1 subunit band intensity detected at 70°C in PANC-1 and BxPC-3 cells was as following in terms of drug treatment: Bortezomib > Elemene >/~ CTRL. Following additional elemene treatment, the denaturation temperature of proteasome β5 and β1 subunit shifted from 70°C to 75°C. CETSA demonstrates elemene protects bortezomib-treated proteasome β5 and β1 against thermal denaturation, which suggests that elemene stabilizes the binding of bortezomib with proteasome in pancreatic cancer cells. Non-covalent binding of elemene with proteasome β1 subunit is only slightly indicated in PANC-1 cells (higher level of β1 subunit in elemene-treated cells than it in CTRL cells), which is probably because weak bonding of elemene is disrupted at such a high degradation temperature of β1 subunit so that it is hard to be observed in CETSA.

These experimental data substantiates above computational modeling and molecular dynamics simulation results, and prove that elemene stabilizes bortezomib binding, and enhances the inhibitory activity of bortezomib toward proteasome, which appears to be the underlying mechanism for their synergistic effects. This “molecular patch” effect of elemene makes the binding site more favorable for bortezomib binding, and therefore increases the proteasome inhibition caused by bortezomib, which consequently result in the dramatically enhanced cytotoxicity of bortezomib in pancreatic cancer cells.

## **Elemene synergizes with bortezomib by enhancing proteasome inhibition, thereby resulting in cell cycle arrest and apoptotic-related cell death in pancreatic cancer cells**

As a potent proteasome inhibitor, bortezomib’s antitumor mechanism of action is through proteasome inhibition to disturb cell cycle regulation and apoptosis process. To determine whether elemene works synergistically with bortezomib in inhibiting cell growth by inducing cell cycle arrest, flow cytometry was performed to analyze the cell cycle distribution upon drug treatment. Cell cycle distribution profiles in **Figure 1o & Figure S8a** show that compared to the control group, elemene or bortezomib at the tested concentration did not induce any cell cycle arrest to neither PANC-1 nor BxPC-3 cells. The fraction of PANC-1 cells in the G2-M phase was 30.6%, 35.4%, and 29.3% after treatment with vehicle, elemene, and bortezomib, respectively. It was substantially increased to 66.0% after treatment with bortezomib plus elemene. The combination of elemene and bortezomib significantly promoted G2/M-phase arrest, accompanied with a decrease in the number of cells in the G0/G1 and S phases. The same trend was also observed in BxPC-3 cells. Taken together, the results suggest that elemene potently enhances bortezomib-induced G2/M-phase arrest in pancreatic cancer cells.

To determine whether elemene works synergistically with bortezomib in inhibiting cell growth by inducing the apoptotic-related cell death, flow cytometry employing Annexin V-FITC/PI staining was performed to analyze the percentage of apoptotic and necrotic cells in PANC-1 and BxPC-3 cells upon drug treatment. As shown in **Figure 1p & Figure S8b**, the percentage of apoptotic cells in bortezomib-treated PANC-1 cells, was significantly increased by ~ 3-4 times with addition of 10 μg/mL elemene. Bortezomib plus elemene caused increases in apoptotic cells which was more obvious in BxPC-3 cells, *i.e.*, ~ 10 times higher than bortezomib group. Simultaneous treatment of elemene and bortezomib induced prominent secondary necrosis after apoptosis in both PANC-1 and BxPC-3 cells. In addition, real-time apoptosis and necrosis of PANC-1 cells in 2D and 3D formats after bortezomib and elemene treatment were monitored by RealTime-Glo Annexin V Apoptosis Assay (**Figure S8c**). Relative luminescence units (RLU) and relative fluorescence units (RFU) represent the magnitude of cell apoptosis and necrosis respectively. In agreement with the results obtained by flow cytometry, compared to single treatment, the combination treatment of elemene and bortezomib significantly induced apoptosis and secondary necrosis in both 2D and 3D PANC-1 cells.

Concordant with the proteomic analysis, computational modeling and the proposed “molecular patch” mechanism, these experimental results show that elemene’s synergistic antitumor effects with bortezomib in pancreatic cancer cells could be predominantly attributed to enhanced proteasome inhibition-associated G2/M-phase arrest and apoptotic-related cell death. In order to confirm the potential regulatory mechanism of elemene in enhancing bortezomib’s effect, the expression levels of some key regulators for cell cycle and apoptosis process including cyclin B1, p53, p21, IkBα, p65 and cleaved-caspase 3 were verified by western blotting. As shown in **Figure 1q**, compared to the bortezomib group, the differential expression of proteasome downstream regulators was observed in elemene and bortezomib combination group, which are comparable with previous studies regarding the effects of proteasome inhibition.[^22-25^](#_ENREF_22) Results of the p21 and cleaved-caspase 3 in cells by WB was also consistent with observation in PANC-1 xenografts tumors by IHC staining (**Figure S9b**). Taken together, all the concatenated data support the role of elemene in producing the synergistic antitumor effects by enhancing proteasome inhibition through the molecular patch mechanism and promoting resultant cell cycle arrest and apoptosis through downstream regulatory proteins.

# Supplementary Figures and Figure Legends

**Figure S1**

**Figure S1**. High throughput screen identifies that elemene enhances the cytotoxicity of bortezomib against PANC-1 cells. (a) NCI approved oncology drug library in dose-response format (left), and concentration-response curve of elemene (right). (b) Concentration-response curve of proteasome inhibitors bortezomib (BZ), ixazomib (IZ) and carfilzomib (CZ) in the absence or presence of elemene (Ele) (n = 3). (c) Images of PANC-1 cells treated with elemene, bortezomib and their combination (4×).

**Figure S2**

**Figure S2**. Elemene does not synergizes with carfilzomib in enhancing the cytotoxicity of carfilzomib against 2D and 3D pancreatic cancer cells. (a, b) Concentration-response curves of elemene (a) and carfilzomib in the absence or presence of elemene (b) in 2D/PANC-1, 2D/BxPC-3, 3D/PANC-1 and 3D/BxPC-3 cells (n = 3). (c) Elemene addition does not augment the inhibitory activity of carfilzomib against cellular proteasome in PANC-1 and BxPC-3 cells (n = 3).

**Figure S3**

**Figure S3**. Top pathway enrichment comparing bortezomib plus elemene vs. bortezomib treatment in PANC-1 cells. (a) Enriched functions in the up-regulated and down-regulated protein groups. (b) Heatmap shows differential expression upon treatments, and the top pathway enrichment analysis between the combination and bortezomib group. Z-score of Log_2_ signal of 156 differentially enriched genes that are associated with proteasome inhibition in three treatment groups was plotted.

**Figure S4**

**Figure S4**. Computational modeling suggests elemene alters inhibitor binding profile by reshaping the ligand binding site of proteasome. (a) Chemical structure of elemene, bortezomib, ixazomib and carfilzomib. (b) X-ray structure of binding conformation of β1-β5-ixazomib and β1-β5-carfilzomib. (c) Modelled binding conformation of β1-β5-ixazomib-elemene and β1-β5-carfilzomib-elemene.

**Figure S5**

**Figure S5**. Molecular dynamics simulations demonstrate the molecular patch mechanism of elemene in enhancing proteasome inhibition. (a) Reaction of bortezomib, ixazomib, and carfilzomib covalently binds to Thr1 of β5 subunit. Atoms and bonds involved in reaction are colored in red. (b) Comparison of histogram distribution of RMSD and distance of bonding atoms with/without elemene binding, in non-covalent binding state, intermediate state and covalent binding state, respectively. The trajectories are aligned based on protein part. For bortezomib and ixazomib, the bonding atom distance is defined as the distance between boron atom and side chain oxygen atom of Thr1 in β5 subunit. For carfilzomib, the distance is defined as the average distance between two bond-forming atoms depicted in panel A. (c) Comparison of MD simulated structure of β1-β5-bortezomib, β1-β5-ixazomib, and β1-β5-carfilzomib with/without elemene binding in non-covalent binding state. Bortezomib, ixazomib and carfilzomib simulated with or without elemene are colored in gold or yellow, respectively.

**Figure S6**

**Figure S6.** Schematic diagram of molecular patch enhanced ligand binding mechanism

**Figure S7**

**Figure S7**. CETSA shows elemene protects bortezomib-treated proteasome β5 and β1 against thermal denaturation, suggesting elemene stabilizes the binding of bortezomib with proteasome. PANC-1 (a) and BxPC-3 (b) cells were treated with elemene and/or bortezomib and subjected to different temperature treatment for 3 mins. Resulting lysates were subjected to western blot analysis for proteasome β1 and β5 detection. GAPDH degrades after 65°C, so thermostable APP-CTFα was used as the loading control.

**Figure S8**

**Figure S8**. Elemene enhances the inhibitory activity of bortezomib toward proteasome, and thus affect downstream regulators of cell cycle and apoptosis process. (a) Cell cycle distribution of 2D/PANC-1 and 2D/BxPC-3 cells upon elemene and bortezomib treatment for 24 h (n = 3). (b) Apoptosis and necrosis after elemene and bortezomib treatment for 24 h determined by flow cytometry (n = 3). (c) Real-time apoptosis and necrosis upon elemene and bortezomib treatment in both 2D/PANC-1 and 3D/PANC-1 cells (n = 3).

**Figure S9**

**Figure S9**. (a) The picture of the xenograft tumors (left) at the endpoint and body weight of tumor-bearing mice (right) from each treatment group during drug treatment (n = 3). (b) Representative IHC staining images and statistics of p21 and cleaved-Caspase 3 in PANC-1 xenografts tumors treated with elemene and bortezomib (40×) (n = 4). The statistics for %positive area of p21 and cleaved-caspase 3 is calculated based on 4 fields per mouse, 4 mice per treatment group.

**References**

1. Eberhardt, J., Santos-Martins, D., Tillack, A.F. & Forli, S. AutoDock Vina 1.2.0: New docking methods, expanded force field, and python bindings. *J Chem Inf Model* **61**, 3891-3898 (2021).

2. Trott, O. & Olson, A.J. AutoDock Vina: Improving the speed and accuracy of docking with a new scoring function, efficient optimization, and multithreading. *J Comput Chem* **31**, 455-461 (2010).

3. Morris, G.M. et al. AutoDock4 and AutoDockTools4: Automated docking with selective receptor flexibility. *J Comput Chem* **30**, 2785-2791 (2009).

4. Case, D.A. et al. AMBER 12. *University of California, San Francisco* (2012).

5. Xu, L., Sun, H., Li, Y., Wang, J. & Hou, T. Assessing the performance of MM/PBSA and MM/GBSA methods. 3. The impact of force fields and ligand charge models. *J Phys Chem B* **117**, 8408-8421 (2013).

6. O'Boyle, N.M. et al. Open Babel: An open chemical toolbox. *J Cheminformatics* **3**, 1-14 (2011).

7. Schauperl, M. et al. Non-bonded force field model with advanced restrained electrostatic potential charges (RESP2). *Commun Chem* **3**, 1-11 (2020).

8. Frisch, M.J. et al. Gaussian 03, rev A.1. *Gaussian Inc., Pittsburgh, PA* (2003).

9. Lu, T. & Chen, F. Multiwfn: a multifunctional wavefunction analyzer. *J Comput Chem* **33**, 580-592 (2012).

10. Kurt, B. & Temel, H. Parameterization of boronates using VFFDT and paramfit for molecular dynamics simulation. *Molecules* **25**, 2196 (2020).

11. Dolinsky, T.J., Nielsen, J.E., McCammon, J.A. & Baker, N.A. PDB2PQR: An automated pipeline for the setup of Poisson–Boltzmann electrostatics calculations. *Nucleic Acids Res* **32**, W665-W667 (2004).

12. Zhou, Y., Zhou, B., Pache, L. & Chang, M. Metascape provides a biologist-oriented resource for the analysis of systems-level datasets. *Nat Commun* **10**, 1523 (2019).

13. Gupta, N. et al. Clinical pharmacology of ixazomib: The first oral proteasome inhibitor. *Clin Pharmacokinet* **58**, 431-449 (2019).

14. Boccadoro, M., Morgan, G. & Cavenagh, J. Preclinical evaluation of the proteasome inhibitor bortezomib in cancer therapy. *Cancer Cell Int* **5**, 18 (2005).

15. Jayaweera, S.P.E., Wanigasinghe Kanakanamge, S.P., Rajalingam, D. & Silva, G.N. Carfilzomib: a Promising proteasome inhibitor for the treatment of relapsed and refractory multiple myeloma. *Front Oncol*, 4683 (2021).

16. Kota, S. et al. A novel three-dimensional high-throughput screening approach identifies inducers of a mutant KRAS selective lethal phenotype. *Oncogene* **37**, 4372-4384 (2018).

17. Li, X. et al. β-elemene sensitizes hepatocellular carcinoma cells to oxaliplatin by preventing oxaliplatin-induced degradation of copper transporter 1. *Sci Rep* **6**, 21010 (2016).

18. Xie, Y. Structure, assembly and homeostatic regulation of the 26S proteasome. *J Mol Cell Biol* **2**, 308-317 (2010).

19. Muz, B. et al. Spotlight on ixazomib: potential in the treatment of multiple myeloma. *Drug Des Devel Ther* **10**, 217 (2016).

20. Rajkumar, S.V., Richardson, P.G., Hideshima, T. & Anderson, K.C. Proteasome inhibition as a novel therapeutic target in human cancer. *J Clin Oncol* **23**, 630-639 (2005).

21. Delport, A. & Hewer, R. A superior loading control for the cellular thermal shift assay. *Sci Rep* **12**, 6672 (2022).

22. Tamura, D. et al. Bortezomib potentially inhibits cellular growth of vascular endothelial cells through suppression of G2/M transition. *Cancer Sci* **101**, 1403-1408 (2010).

23. Shah, S.A. et al. 26S proteasome inhibition induces apoptosis and limits growth of human pancreatic cancer. *J Cell Biochem* **82**, 110-122 (2001).

24. Nawrocki, S.T., Sweeney-Gotsch, B., Takamori, R. & McConkey, D.J. The proteasome inhibitor bortezomib enhances the activity of docetaxel in orthotopic human pancreatic tumor xenografts. *Mol Cancer Ther* **3**, 59-70 (2004).

25. Ling, Y.H. et al. Mechanisms of proteasome inhibitor PS-341-induced G2-M-phase arrest and apoptosis in human non-small cell lung cancer cell lines. *Clin Cancer Res* **9**, 1145-1154 (2003).
